# Importance of Energy, Dietary Protein Sources, and Amino Acid Composition in the Regulation of Metabolism: An Indissoluble Dynamic Combination for Life

**DOI:** 10.3390/nu16152417

**Published:** 2024-07-25

**Authors:** Giovanni Corsetti, Evasio Pasini, Tiziano M. Scarabelli, Claudia Romano, Arashpreet Singh, Carol C. Scarabelli, Francesco S. Dioguardi

**Affiliations:** 1 Division of Human Anatomy and Physiopathology, Department of Clinical and Experimental Sciences, University of Brescia, 25023 Brescia, Italy; cla300482@gmail.com; 2 Italian Association of Functional Medicine, 20855 Lesmo, Italy; evpasini@gmail.com; 3 Department of Clinical and Experimental Sciences, University of Brescia, 25023 Brescia, Italy; 4 Holy Cross Medical Center, Taos, NM 87571, USA; tscarabelli@hotmail.com (T.M.S.); chenscarabelli@hotmail.com (C.C.S.); 5 School of Osteopathic Medicine, Campbell University, Lillington, NC 27546, USA; a_singh1001@email.campbell.edu; 6 Nutri-Research s.r.l., 20127 Milan, Italy; fsdioguardi@gmail.com

**Keywords:** energy, entropy, protein synthesis, amino acids, autophagy, AMPK, mTOR

## Abstract

Purpose. This paper aims to present a unique perspective that emphasizes the intricate interplay between energy, dietary proteins, and amino acid composition, underscoring their mutual dependence for health-related considerations. Energy and protein synthesis are fundamental to biological processes, crucial for the sustenance of life and the growth of organisms. Methods and Results. We explore the intricate relationship between energy metabolism, protein synthesis, regulatory mechanisms, protein sources, amino acid availability, and autophagy in order to elucidate how these elements collectively maintain cellular homeostasis. We underscore the vital role this dynamic interplay has in preserving cell life. Conclusions. A deeper understanding of the link between energy and protein synthesis is essential to comprehend fundamental cellular processes. This insight could have a wide-ranging impact in several medical fields, such as nutrition, metabolism, and disease management.

## 1. Introduction

This paper is designed to articulate a particular viewpoint, rather than present a narrative review or introduce new findings. Our intent is to provide an overall understanding of the interconnectedness of energy metabolism, protein synthesis, and amino acid (AA) availability, viewed as a dynamic system. The concept of this dynamic equilibrium was first proposed by the Nobel laureate George Hoyt Whipple in the 1930s, suggesting that tissue proteins exist in a state of flux with circulating plasma proteins [1].

In 1942, Rudolf Schoenheimer stated that “*not only the fuel, but also the structural materials, are in a steady state of flux*”, thereby introducing the concept of “*The dynamic state of body constituents*” [2]. This concept, while intuitive, has not garnered the attention it merits in the medical field. Often, the focus remains on mere information gathering rather than on deepening our understanding [3]. In essence, information is readily available and accessible, but knowledge is gained through the processing and comprehension of this information. In medical practice, both are vital for delivering high-quality patient care.

Before delving into the subject matter, we deem it essential to revisit some fundamental concepts.

The primary objective of cells is to generate and utilize energy in the form of adenosine triphosphate (ATP) for physiological functions such as molecule synthesis, movement, and duplication. However, the second law of thermodynamics makes these tasks more challenging than they initially appear. This law introduces the concept of order and disorder, which is intricately linked to the amount of energy involved. As the available energy in a cell decreases, the cell becomes more random, a state referred to as entropy. High entropy equates to increased disorder and low energy. It is understood that a cell must maintain a high degree of order to survive, necessitating a substantial amount of energy to sustain a state of low entropy and remain alive [4]. From this viewpoint, it can be postulated that a cell must continuously balance energy production and expenditure to maintain equilibrium. This balance between catabolic and anabolic processes is crucial for cell survival.

Therefore, energy production and protein synthesis are deeply interconnected processes at the heart of cellular biology, being highly interdependent and mutually regulated [5,6]. Regrettably, despite the widespread understanding of each aspect of these processes, they are seldom regarded as deeply interconnected elements that have the potential to significantly impact an organism’s health.

Therefore, the objective of this paper is to provide not just a summary of the information available, but also an in-depth comprehension of the dynamic interaction between energy, protein synthesis and sources, and AA composition. This will be achieved by presenting an integrated view of the processes that regulate this intricate combination.

## 2. Energy Metabolism

Metabolic systems, ranging from organisms to cellular organelles and biochemical pathways, often exist in a steady state. In this state, reaction rates and concentrations of metabolic intermediates remain constant or fluctuate within narrow values. Factors such as the characteristics, kinetics, and activities of enzymes, temperature, and concentrations of endogenous and exogenous molecules shape this steady state. Typically, for a given set of parameters, there exists a single steady state, which is upheld by the system variables. Metabolites directly affect the reaction rates. If the steady state is disrupted, the variables involved react to reestablish it [7]. Energy-producing pathways often intersect and even intertwine with biomass formation pathways [8].

Energy in biological systems primarily derives from the catabolism of macronutrients such as carbohydrates, lipids, and proteins, triggered by specific stimuli. These nutrients undergo a series of enzymatic reactions to produce ATP, the universal energy currency of cells.

In eukaryotic cells, energy production unfolds in a three-step process that is tightly interconnected: (1) anaerobic glycolysis in the cytosol; (2) the citric acid cycle, alias, the Krebs cycle; (3) aerobic oxidative phosphorylation in the mitochondria. It is crucial to note that cells cannot store ATP easily, necessitating its continuous production to meet their needs and keep their metabolism in a constant state of flux [9].

Recent research on T cell energy and protein metabolism shows that cellular metabolic pathways involved in both protein antibody synthesis and energy production are activated following specific antigen receptor stimulation [10,11]. In this context, glucose metabolism significantly contributes to ATP synthesis. Both the anaerobic glycolytic cycle and aerobic mitochondrial respiration are enhanced in activated T cells. The expression of glucose transport proteins and glycolytic enzymes is swiftly induced. Concurrently, the mitochondrial Krebs cycle flow and mitochondrial oxidative capacity and biogenesis are increased [12]. Notably, the mitochondrial function is also fueled by AAs, which supply energetic substrates and generate crucial Krebs cycle intermediates to maintain an active mitochondrial function and ATP production. This observation further confirms the close link between energy dynamics and protein metabolism. In addition, recent studies demonstrated that a specific blend of essential AAs (EAAs) stimulates mitochondrial biogenesis and function, thereby increasing the number of these cytoplasmic organelles and strengthening energy production [13,14].

## 3. Protein Synthesis and Regulatory Mechanisms

Protein synthesis, a critical process in all biological systems, is vital for sustaining life and facilitating organism growth and development [5]. It is worth noting that protein synthesis is an energy-demanding process, consuming a minimum of four ATP molecules for each peptide bond. Therefore, the synthesis of a standard protein, such as albumin, necessitates over 2900 ATP molecules. These ATP molecules are generated from the mitochondrial oxidation of approximately 60–70 glucose molecules, assuming that the cell metabolism operates with low entropy [15]. This process is regulated by intricate metabolic mechanisms. Indeed, as previously discussed, both energy production and protein synthesis must be stringently regulated to meet the cellular and life demands.

There are 20 proteinogenic AAs, classified as essential (E) or non-essential (NE), a categorization that, despite certain limitations, is easy to understand and apply. Already since the 1940s, it was established that the only protein derivatives essential and sufficient to maintain health and nitrogen balance were EAAs [16]. Therefore, protein synthesis in cells requires a sufficient supply of AAs, in particular EAAs [17], and an adequate amount of energy (ATP). Indeed, EAAs must be regularly replenished through diet, as mammals cannot synthesize them autonomously, except to a limited extent that is incompatible with life. Non-essential AAs (NEAAs), while abundantly supplied by food, can also be synthesized by the body as needed, starting from EAAs. Thus, the availability of all EAAs in sufficient amounts is the limiting factor in protein synthesis, as previously reported [18,19]. When conditions are met, protein synthesis can optimally proceed to construct proteins, be they filamentous, globular, or enzymes.

Protein synthesis is a complex, energy-intensive process that involves multiple steps, including transcription, translation, and post-translational modifications. The primary players in these key regulatory mechanisms include the following.

### 3.1. Energy Sensors

It is well established that energy is released when ATP is converted to adenosine diphosphate (ADP). Additional energy is released when a phosphate group is removed from ADP to form adenosine monophosphate (AMP). When the cell consumes a large amount of energy, and energy availability decreases (i.e., during protein synthesis), the ATP/AMP ratio decreases, thereby stimulating AMP-activated protein kinase (AMPK). AMPK is an energy sensor that curbs chemical synthesis in response to the cellular energy levels.

AMPK is a serine/threonine protein kinase complex composed of a catalytic subunit (α), a scaffold subunit (β), and a regulatory subunit (ϒ), each with a distinct role in regulating allosteric enzymatic activities. Interestingly, when AMP binds to AMPK, it triggers a significant conformational change, inducing the formation of a loop that inhibits phosphatase action. Conversely, when AMPK binds to ATP, it rotates by approximately 180°, enabling its dephosphorylation and thus its deactivation [20].

**Figure 1:**
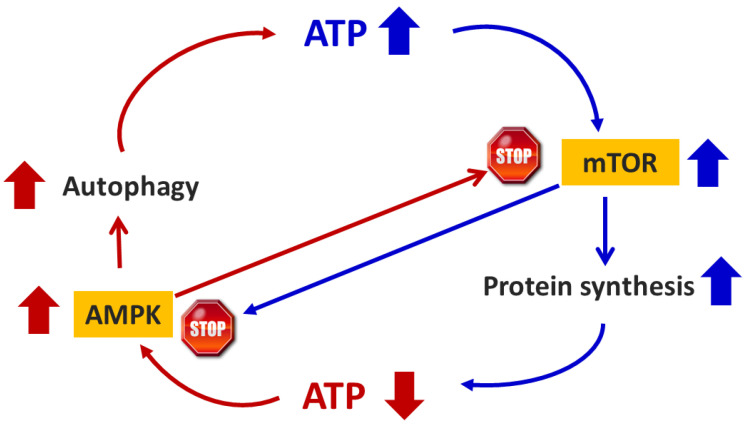
Schematic representation of the interaction between AMPK and mTOR and their influence on energy (ATP) production.

Hence, AMPK is an energy sensor that regulates several metabolic processes, including protein synthesis, in response to the cellular energy levels. AMPK manages the cellular energy availability by activating the flow of substrates towards energy-producing metabolic pathways and deactivating energy-consuming ones. Specifically, AMPK inhibits the production of fatty acids, cholesterol, and triglycerides but promotes the cell fatty acid uptake and oxidation. It also stimulates glucose uptake in cells by activating glucose transporters. Moreover, it triggers enzymes of glycolysis and inhibits glycogen synthase [21]. AMPK also suppresses energy-consuming protein synthesis and stimulates autophagy (AUT) by binding to a specific unit of the cytoplasmic signaling enzymatic system named mammalian target of rapamycin (mTOR) (Figure 1).

AUT is a self-degradative process that has long been recognized as fundamental for balancing energy sources during times of metabolic stress. It is a catabolic adaptive response that aids survival by breaking down cellular components to maintain the availability of molecules essential for cellular metabolism, as observed in mice between birth and suckling [22].

It is now documented that AMPK regulates AUT through well-known mechanisms. AMPK activates AUT by down-regulating mTOR activities and/or, more significantly, stimulating the enzymatic complex Unc-51-Like Kinase 1 (ULK1), which is crucial for AUT initiation. Indeed, AMPK-mediated phosphorylation of ULK1 stimulates autophagosome biogenesis, the first step of AUT. Additionally, AMPK regulates the subsequent step of AUT, the fusion of the outer membrane of an autophagosome and a lysosome to form an autolysosome. This process transports the encapsulated materials to the lysosomal lumen, where specific enzymes break down the material, making individual molecules of AAs available for protein synthesis and/or mitochondrial respiration and releasing lipids and carbohydrates critical for energy production [23].

However, it is important to emphasize that, under homeostatic conditions, AUT is a natural metabolic process that, through lysosome-dependent regulated mechanisms, allows the cell to eliminate unnecessary and/or dysfunctional structures, including proteins. This process facilitates the recycling and/or increased availability of fundamental macromolecules to support cellular metabolism, both for energy and for synthetic purposes [24].

On the contrary, during acute and/or chronic stress situations, such as starvation or catabolic stimuli, AUT can serve as an adaptive temporary response contributing to cell survival by maintaining cytosolic molecules, primarily EAAs, which can then be recycled for new protein synthesis and/or funneled through energy production cycles.

For instance, in cases of intracellular nutrient shortage secondary to starvation/growth factor withdrawal or impaired ATP synthesis in the setting of ischemia, AUT can serve as an adaptive response promoting cell survival, either by purging the cell of damaged organelles or by generating the intracellular building blocks required to maintain vital functions, which ultimately results in ATP production, protein synthesis, and improved cell survival with recovery of myocyte function [25,26,27]. Conversely, under more extreme conditions, AUT may also promote cell death through excessive self-digestion and degradation of essential cellular constituents [28,29].

### 3.2. Amino Acid Availability

Both EAAs and NEAAs are derived from the digestion of dietary proteins. However, the efficiency of protein digestion and absorption of proteins as AAs decreases with aging [30].

The body can synthesize NEAAs from EAAs when metabolically necessary [5]. The presence of EAAs in the cytosol plays a crucial role in regulating cell metabolism, including protein synthesis and energy homeostasis [31]. Notably, a specific pool of EAAs, known as branched-chain amino acids (BCAAs), particularly leucine, has a primary role in modulating the function of proteins involved in both global mRNA translation and the selection of specific mRNAs for translation through mTOR activation [32].

Adequate amounts of cytosolic AAs for protein synthesis are also available through alternative mechanisms, such as the degradation of intracellular proteins by AUT [33]. Interestingly, apart from being the building blocks of proteins, AAs, particularly EAAs, have been documented to have other significant metabolic regulatory functions. Thus, they have recently been defined as “metabokines”, which are molecules also capable of influencing many cellular metabolic pathways. AAs regulates several energy metabolism pathways in multiple tissues, including fatty acid β-oxidation, mitochondrial oxidative phosphorylation, lipolysis, glycolysis, and gluconeogenesis [34]. In addition, data show that AAs regulate multiple processes related to gene expression, including the modulation of the function of proteins that mediate mRNA translation [32,35]. Moreover, it has been demonstrated that a stoichiometrically balanced mixture of EAAs influences mitochondrial energy production, not only providing fuel and/or Krebs cycle intermediates, but also stimulating the production of NO from eNOS, which favors the expression of enzymes responsible for mitochondrial biogenesis, such as Peroxisome Proliferator-Activated Receptor-Gamma Coactivator (PGC-1α) and Tfam, with a consequent increase in mitochondria number [13,14] and mitochondrial bioenergetics [36].

### 3.3. mTOR Signaling

The mammalian target of rapamycin (mTOR) pathway is a cytoplasmic signaling pathway that controls cell growth and global metabolism, including protein and energy synthesis, in response to nutrient availability, cellular energy, and stress.

mTOR is a complex serine/threonine protein kinase in the PI3K-related kinase (PIKK) family and forms the catalytic subunit of two distinct protein complexes: mTOR Complex 1 (mTORC1) and mTOR Complex 2 (mTORC2). Both mTORC1 and mTORC2, each containing both common and unique subunits, play significant roles in cellular metabolism. mTORC1 includes Raptor, whereas mTORC2 contains Rictor, Protor, and mSin1 (also known as MAPKAP1) [37]. Despite being inhibited by Deptor, either mTORC1 or mTORC2 can deregulate Deptor expression, allowing the mTOR enzymatic complex to influence cell metabolism in various ways, depending on a cell’s metabolic needs [38]. Additionally, mTOR responds to the cytoplasmic energy amount, as sensed by AMPK. When the energy levels are high, mTOR stimulates potential protein synthesis and cell growth. Conversely, when the ATP/AMP ratio decreases, mTOR influences catabolic processes such as AUT [39,40,41]. These complex interactions increase mTOR-dependent control and fine regulation of anabolic or catabolic pathways.

In addition to energy production, mTOR also modulates protein synthesis. For instance, it regulates translation factors like eIF4EBPs and promotes the phosphorylation of the ribosomal subunit S6, which is crucial for protein synthesis. mTOR also acts as a transcriptional regulator of mitochondrial functions, stimulating genes such as PGC-1α and Estrogen-Related Receptor-α (ERR-α), leading to increased mitochondrial respiration in skeletal muscle tissue and many cell lines. The impact of mTOR on PGC-1α involves ying yang-1 (YY1), a member of the GLI-Kruppel class of zinc finger proteins that acts as a transcriptional regulator [42,43].

Notably, evidence shows that EAAs also activate the mTOR pathway. Like insulin, specific EAAs activate protein synthesis, stimulate ribosome synthesis, and suppress AUT by stimulating mTOR-dependent metabolic pathways in muscles. However, unlike insulin, certain EAAs do not stimulate mTOR via phosphoinositide 3-kinase and Akt but indirectly activate the TSC1/2-Rheb complex through the small guanosine triphosphatase Rheb protein. In detail, TSC2 is a guanosine triphosphatase-activating protein acting on Rheb that, through still unknown mechanisms, either directly or indirectly regulates mTOR activity [44,45,46].

Interestingly, we demonstrated that a special mixture of EAAs influences mTOR signaling, inducing muscle protein synthesis in both skeletal and cardiac muscles of young and elderly healthy sedentary and trained rats [13]. However, it is important to emphasize that these nutrient- and energy-sensitive pathways form a complex network of reactions that influence each other and are likely organ-dependent. Further studies are needed to understand these complex phenomena in more detail.

### 3.4. Transcription Factors

Conditions such as AA deprivation and mitochondrial respiratory chain dysfunction, leading to reduced energy production, activate transcription factors like Activating Transcription Factor 4 (ATF4). ATF4 serves as a stress integrator for nutrient and energy signals, modulating the gene expression of protective protein chaperones like GPR78/BiP, which regulate protein refolding, enzymes, and antioxidants such as heme oxygenase. Moreover, it has been demonstrated that ATF4 significantly interacts with mTOR and regulates the expression of genes involved in AUT, such as ULK1 [47,48,49,50].

**Figure 2:**
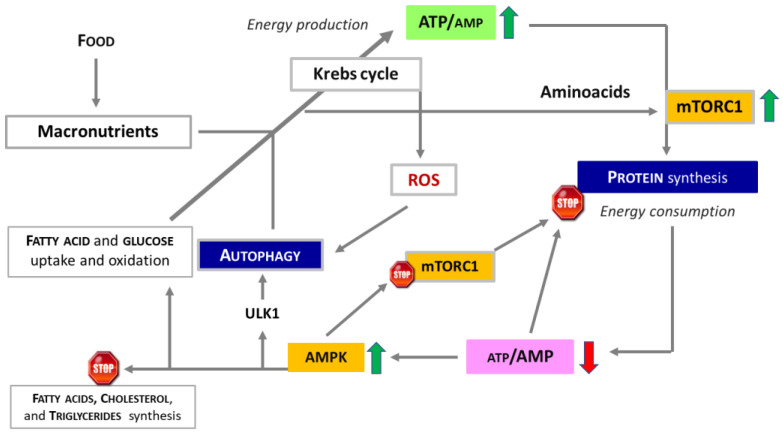
Energy levels (amount of ATP) regulate the levels of both protein synthesis and autophagy. The production of ATP through the Krebs cycle provides the energy necessary for protein synthesis by activating mTORC1. However, it also produces reactive oxygen species (ROS), which have been shown to induce autophagy. Low energy levels resulting from the consumption of ATP for the construction of thousands of peptide bonds necessary for protein synthesis activate AMPK, which in turn inhibits mTORC1 and activates autophagy, providing substrates to support ATP production. ULK1, Unc-51-Like Kinase 1.

The synergistic interplay between food, energy production, protein synthesis, AA availability, and AUT ensures the maintenance of cellular homeostasis and overall health, as depicted in Figure 2.

## 4. Dietary Proteins: Quality and Sources

### 4.1. The Importance of Protein Quality

Proteins play an indispensable role in all living organisms’ functions and are found in every tissue. In humans, proteins constitute around 15% of the body mass, with over 60% being contractile proteins, primarily concentrated in muscle tissues. Therefore, proper protein nutrition is crucial for maintaining bodily functions and, ultimately, health.

However, not all dietary proteins are created equal. The quality of proteins consumed, not just the quantity, is of utmost importance. This concept is often overlooked, especially when formulating protein supplements. The quality of a protein is determined by the presence of all EAAs in adequate quantities, as well as by its digestibility and absorption, all of which increase its utility for the body. High-quality proteins can be found in both animal and vegetable foods, such as milk, eggs, salmon, lean meats, and soy proteins. This leads us to a crucial qualitative concept: the biological value (BV) of proteins. The BV is a multifactorial measure that considers the quantity, quality, and mutual relationship of the EAAs present in food proteins. Essentially, it describes a protein quality and the constructive potential of the AAs contained within it. BV can also be defined as the ratio between retained nitrogen and absorbed nitrogen, minus the amount eliminated through sweat, feces, and urine.

### 4.2. Plant or Animal Protein Sources and Intake

A recent systematic review and meta-analysis of prospective cohort studies suggested that plant proteins are associated with a lower risk of overall mortality, including from cardiovascular diseases and tumors [51]. However, this situation changes with age. For older adults, plant proteins may not be the best solution. A recent study among adults aged fifty-one and older showed that doubling the plant protein amount in the diet resulted in a 22% decrease in total protein intake, indicating malnutrition [52]. These data suggest that increasing plant-based foods while reducing animal products could have negative health effects on the population aged over fifty-one, both males and females.

Furthermore, in adults aged over 71 years, doubling the plant protein intake resulted in an average protein intake of only about 0.8 g/kg/day of body weight [52]. Among women over 71, 33% could not meet their average daily protein needs. In elderly males, even though the protein intake could barely meet the theoretical daily recommendation, it still failed to meet the daily intake of 1.0–1.2 g/kg of protein recommended by the PROT-AGE Study Group of the European Union Geriatric Medicine Society (EUGMS). The efficiency of plant protein varies greatly depending on the protocol applied, making it difficult to provide clear recommendations on how plant proteins should be incorporated into specific dietary patterns [53]. On the other hand, doubling the consumption of dairy products easily met the recommended protein levels. Therefore, for older adults, increasing the dairy product intake may help achieve the appropriate daily protein nutritional level (approximately 1.2 g/kg), which aligns with the growing consensus that older adults need to consume more proteins to maintain health and quality of life [52].

### 4.3. Insect Proteins

Historically, insects have been consumed and institutionally accepted as food in many regions due to their sufficient nutritional value for humans [54,55,56]. Recently, amid concerns over potential food resource shortages, various alternative food sources have been proposed for industrialized countries, with insects garnering significant interest [57]. The resurgence of insects as a food source is linked to their nutritional, environmental, and economic value [58,59]. The nutritional value of insects can vary based on factors such as diet, developmental stage, sex, species, growth environment, and analysis methods [59,60,61]. However, there is a consensus that insects are extremely rich in proteins, fats, and vitamins [62]. On average, the protein content of edible insects ranges from 35% to 60% in dry weight or from 10% to 25% in fresh weight [63,64]. These values are higher than those for plant protein sources like cereals, soy, and lentils [55]. Some insect species even appear to offer more protein than chicken meat and eggs [65]. However, the digestibility of insect proteins is highly variable due to the presence of chitin in the exoskeleton, which is nearly indigestible for humans [64,66]. If chitin is removed, digestibility appears to increase, ranging from 77% to 98% [67]. The current literature primarily focuses on extraction and purification techniques, with a lack of scientific data regarding the actual utility of these proteins.

### 4.4. Protein Intake and Utilization

Recently, a pilot study in humans found that the intake of pea- and whey-derived proteins (both proteins providing EAAs in noticeable amounts, still EAAs/NEAAs <<0.9) produced comparable results in body composition, muscle volume, force production, daily performance, and strength after 8 weeks of high-intensity functional training [68]. However, the current studies are limited and often yield conflicting results. Nonetheless, plant proteins can still offer nutritional benefits, even with qualitative limitations that render them inferior to animal proteins. In terms of elderly nutrition, while guidelines still recommend the same protein amount throughout adulthood (0.8 g/kg/day~56 g/day for males and ~46 g/day for females) [69], many recent studies recommend higher protein amounts for those over 65. This suggests that a daily protein intake of at least 1.0–1.2 g/kg is beneficial for general health, recovery after illness, and functional status maintenance, especially in the elderly [70]. An even higher protein intake (1.2–1.5 g/kg/day) is crucial for those with acute or chronic diseases, while individuals with serious illnesses, acute injuries, or severe malnutrition may require a protein intake of at least 2.0 g/kg [71], due to the presence of hypercatabolic metabolism. Unfortunately, proteins are never fully utilized. Protein digestion typically takes from 1 to 2 h, and only about 40–70% of the AAs that make up proteins are assimilated [30]. The unabsorbed ratio of alimentary proteins constitutes nitrogenous waste that must be eliminated by the kidneys and liver. This is why a diet excessively high in protein can overload these organs over time, potentially compromising their function.

## 5. Protein Turnover and Requirements

Even in a state of rest and in healthy individuals, proteins, in all their forms and functions, undergo a continuous process of degradation and synthesis. This turnover allows the body to replace worn-out molecules and maintain optimal function. For instance, heart proteins are typically renewed every 30 days, and muscles degrade 250–350 g of protein per day, necessitating replenishment. Skeletal muscle, which constitutes approximately 40% of the body weight, contains 50–75% of all proteins in the human body [72]. The total body protein turnover, which includes simultaneous processes of protein synthesis and breakdown, accounts for approximately 20% of resting energy expenditure. It is estimated that about 1–2% of the total skeletal muscle mass undergoes turnover [72]. Consequently, despite its primary role of converting chemical energy into mechanical energy for movement, muscle also plays a significant role in metabolism. It acts as a storage site for an energetic substrate (glycogen), a nitrogen donor, and a source of gluconeogenic molecules and fuels (amino acids), which are essential during physical activities and/or instances of malnutrition, starvation, injuries, and chronic diseases [73].

### 5.1. Physical Activity and Hypercatabolic Syndrome

Physical activity is beneficial at any age. Data show that regular movement can enhance the body’s ability to synthesize proteins, whereas a sedentary lifestyle can diminish this ability and affect the absorption of certain nutrients. Consequently, healthy adults who engage in moderate to vigorous physical activity require more protein (1.3–1.6 g/kg/day) to boost their muscle mass and physical strength compared to their sedentary counterparts [74]. Notably, it is recommended to increase the total daily protein intake to at least 1.2 g/kg in healthy older people because aging negatively influences the protein metabolism due to intrinsic malnutrition and/or catabolic stimuli caused by an aging-induced altered inflammation process, termed immunosenescence [75].

Indeed, conditions such as aging, chronic and/or autoimmune diseases, injuries, and tumors increase catabolic stimuli due to the production of catabolic inflammatory molecules (i.e., cytokines and hormones), leading to the Hypercatabolic Syndrome (HS). The HS significantly increases whole-body metabolism, thereby increasing energy consumption and disrupting the balance between anabolic and catabolic stimuli. This imbalance results in the breakdown of muscle contractile proteins and circulating visceral proteins and the release of AAs. In these metabolic conditions, the released AAs are deaminated, and the resulting carbon skeletons are used to produce energy and other metabolic intermediates necessary to meet the increased metabolic demands. In this context, the role of skeletal muscle and circulating visceral proteins extends beyond ensuring posture maintenance and locomotion and transporting molecules or atoms [76].

The biochemical consequence of HS is a protein disarrangement, which clinically manifests through symptoms such as sarcopenia, hypoalbuminemia, anemia, infections, and fluid compartmentation alterations. These symptoms result in increased hospitalization and morbidity for the patient [76]. In these metabolic conditions, the synergy between adapted physical activity and protein nutrition becomes crucial. Emphasis should be placed on providing high-biological-value proteins and, most importantly, on ensuring the intake of all EAAs [74].

Any increase in the body’s metabolic demand necessitates a greater nitrogen (protein) nutritional intake, where all EAAs must be present in the correct quantities. Studies have shown that oral supplementation with an EAAs mixture in a stoichiometric ratio supports the body metabolism in both aging and chronic hypercatabolic diseases such as diabetes, cardiomyopathies, and tumors [6,45,76,77].

### 5.2. The Limits of Protein Intake

While some healthy adults, especially athletes, can tolerate a higher protein intake, a review suggests that consuming more than 2 to 2.5 g/kg/day of protein (approximately 25% of the energy needs) might be excessive [78]. Occasional excess protein consumption could lead to transient gastrointestinal issues. However, a consistently high protein intake over the long term may contribute to digestive, renal, and vascular abnormalities [74]. Moreover, a high consumption of animal protein can increase the risk of cardiovascular death [79,80]. Therefore, individuals of all ages need a balanced diet that includes a variety of macro- and micronutrients. Consuming a diet that is too high in protein, particularly animal protein, can have negative health effects. The key is to maintain a balanced intake of all nutrients, not just proteins, for overall health and wellbeing.

Recent research has revealed a mechanism in which a high protein intake, through an increase in plasma leucine, leads to mTORC1-mediated inhibition of monocyte/macrophage autophagy, subsequently causing atherogenesis. This discovery has significant clinical and public health implications. Protein intake at any level above the minimum recommended daily intake (0.8 g/kg/day) is generally considered safe and acceptable and has gained popularity. However, a high protein and/or leucine intake should be approached with caution [80]. As such, the daily protein intake must be carefully evaluated and tailored to the patient’s metabolic conditions.

## 6. The Significance of EAA Supplementation

Cells frequently adapt their metabolic strategies under conditions of nutrient deprivation to sustain their survival and growth. Therefore, adequate protein nutrition is crucial at various ages and in different physiological states. Years ago, a study in humans demonstrated several key findings related to AA administration. Firstly, the absolute increase in energy expenditure is dose-dependent and does not appear to reach a plateau. Secondly, this increase is positively correlated with AA-induced protein synthesis. Lastly, the thermic effect is not dependent on the dose of AAs administered [81]. These findings underscore the complex interactions between protein intake, metabolic responses, and physiological conditions.

Quantifying the dietary AA intake is crucial, as both exogenous and endogenous AAs contribute to protein synthesis. While some studies reported habitual AA intakes, none assessed adherence to the Dietary Reference Intakes for each EAA [82]. Unfortunately, all dietary proteins, including those with a high BV, have an EAAs/NEAAs ratio of less than 1, meaning that are always in excess. Typically, the EAAs/NEAAs ratio in a food protein is approximately 30/70, requiring organisms to consume large quantities of NEAAs to meet the daily need for all EAAs in adequate amounts.

Recent studies in experimental models showed that a diet with moderate EAA deficiency significantly reduced survival as a function of EAA concentration, while an EAA excess increased survival [17,18]. However, under normal conditions, about 70% of the EAAs obtained from muscle protein degradation are reincorporated into other muscle proteins. Unfortunately, the efficiency of this process can only be partially increased. Consequently, supplementing EAAs by providing exclusively single BCAAs, although required in greater quantities, cannot support or increase the rate of muscle protein synthesis, because the limited availability of other EAAs quickly becomes the limiting factor [83]. Therefore, an anabolic state cannot occur without the availability of all EAAs in adequate quantities.

The positive effect of supplementation with a mixture of all single EAAs in stoichiometric ratio, according to the human metabolic needs, has been observed in numerous experimental conditions [84,85,86], including chemotherapy [87,88]. This effect is based on the stimulation of anabolism through the activation of eNOS, leading to mitochondrial biogenesis and the reduction of reactive oxygen species (ROS) [13], as well as mTORC1 activation, resulting in increased protein synthesis [89]. Furthermore, free EAAs do not need to be digested; so, they are rapidly absorbed and quickly available in the circulation to support the cell metabolism [90].

Recent studies indicated that certain metabolites and nutrients, including AAs, that are not classified as vitamins, cytokines, or hormones, can regulate fundamental metabolic cell pathways. These bioactive metabolites have been termed metabokines [34]. This new understanding of metabokines expands our knowledge of the complex interactions within cellular metabolism and may open new avenues for therapeutic interventions.

Emerging evidence suggests that EAAs act as metabokines, influencing the metabolism of not only healthy cells but also diseased ones, including tumor cells [35].

For example, diets that selectively restrict all NEAAs have been shown to increase the life expectancy of mice with colon cancer, suggesting a potential therapeutic role in humans [91]. Specific dietary patterns with various food energy sources, including some AAs, can induce tumor cells into a state of non-proliferative senescence [92]. Additionally, supplementation with leucine has been shown to counteract cancer-induced cachexia [93]. A thorough review of the existing literature on the interplay between metabolism and cell death in tumorigenesis underscores the significant role that various metabolic processes play in either promoting or inhibiting cell death. This influence is not only through direct stimuli causing stress and cell death, but also through impacting key regulators of different cell death processes.

The tumor microenvironment is also implicated in the metabolic regulation of cell death, suggesting that modulating cancer cell metabolism could be a viable and effective strategy to regulate tumorigenesis [94]. The characterization of metabolic reprogramming in the tumor microenvironment is becoming increasingly crucial in cancer research and patient care [95]. This emerging focus could potentially pave the way for innovative therapeutic approaches in cancer treatment.

Another example concerns heart diseases. Cardiac cachexia continues to pose a significant clinical challenge in the management of patients with heart failure. This condition is characterized by unintentional weight loss, resulting from catabolism, a metabolic state where fat and skeletal muscle mass are broken down to fulfill the body’s energy requirements. Cardiac cachexia is prevalent in patients with advanced heart failure and is independently associated with mortality [96].

Studies have revealed that patients with chronic heart failure exhibit reduced arterial AA levels, which restricts the supply and availability of AAs to the heart. This is directly correlated with clinical disease severity and left ventricular dysfunction [97].

These findings underscore the need for rigorous nutritional monitoring and dietary education for patients with chronic heart failure and all patients in a hypercatabolic state. This should be supplementary to standard dietary advice for these individuals. The objective is to ensure an optimal nutritional status that supports the overall health and potentially mitigates the progression of cardiac cachexia.

These studies confirm that AAs and their metabolites can influence the cellular metabolism by acting as metabokines capable of exerting epigenetic control of metabolic pathways in both normal and pathological cells. This evidence opens new avenues for the therapeutic use of AAs.

## 7. Conclusions

Life’s intricate machinery depends on the dynamic flux and the interrelated influences among energy, protein synthesis, and the availability of AAs. These elements must work in synergy and maintain a mutual balance. A comprehensive understanding of these processes provides valuable insights into the molecular basis of various diseases and presents potential targets for therapeutic intervention, emphasizing their inseparable dynamic combination for life’s sustenance. Emerging findings support the notion that supplementation with an EAA mixture containing all single EAAs in stoichiometric ratio could be clinically significant in hypercatabolic and/or malnourished patients.
